# What’s the cut-point?: a systematic investigation of tau PET thresholding methods

**DOI:** 10.1186/s13195-022-00986-w

**Published:** 2022-04-05

**Authors:** Alexandra J. Weigand, Anne Maass, Graham L. Eglit, Mark W. Bondi

**Affiliations:** 1 Joint Doctoral Program in Clinical Psychology, San Diego State University/University of California, San Diego, USA; 2 German Center for Neurodegenerative Diseases, Magdeburg, Germany; 3 Research Service, VA San Diego Healthcare System, San Diego, USA; 4 Department of Psychiatry, University of California, San Diego, USA; 5 Neuropsychological Assessment Unit, University of California San Diego School of Medicine, VA San Diego Healthcare System (116B), 3350 La Jolla Village Drive, San Diego, CA 92161 USA

**Keywords:** Tau, Positron emission tomography, Methods, Alzheimer’s disease, Review

## Abstract

**Background:**

Tau positron emission tomography (PET) is increasing in popularity for biomarker characterization of Alzheimer’s disease (AD), and recent frameworks rely on tau PET cut-points to stage individuals along the AD continuum. Given the lack of standardization in tau PET thresholding methods, this study sought to systematically canvass and characterize existing studies that have derived tau PET cut-points and then directly assess different methods of tau PET thresholding in terms of their concurrent validity.

**Methods:**

First, a literature search was conducted in PubMed to identify studies of AD and related clinical phenotypes that used the Flortaucipir (AV-1451) tau PET tracer to derive a binary cut-point for tau positivity. Of 540 articles screened and 47 full-texts reviewed, 23 cohort studies met inclusion criteria with a total of 6536 participants. Second, we derived and compared tau PET cut-points in a 2 × 2 × 2 design that systematically varied region (temporal meta-ROI and entorhinal cortex), analytic method (receiver operating characteristics and 2 standard deviations above comparison group), and criterion/comparison variable (amyloid-beta negative cognitively unimpaired or cognitively unimpaired only) using a sample of 453 older adults from the Alzheimer’s Disease Neuroimaging Initiative.

**Results:**

For the systematic review, notable variability in sample characteristics, preprocessing methods, region of interest, and analytic approach were observed, which were accompanied by discrepancy in proposed tau PET cut points. The empirical follow-up indicated the cut-point derived based on 2 standard deviations above a either comparison group in either ROI best differentiated tau positive and negative groups on cerebrospinal fluid phosphorylated tau, Mini-Mental State Examination score, and delayed memory performance.

**Conclusions:**

Given the impact of discrepant thresholds on tau positivity rates, biomarker staging, and eligibility for future clinical treatment trials, recommendations are offered to select cut-point derivations based on the unique goals and priorities of different studies.

## Introduction

Tau neurofibrillary tangles (NFTs), along with amyloid (Aβ) plaques, represent the two defining pathologic hallmarks of Alzheimer’s disease (AD; [1]). Although Aβ has predominated AD research for the past 30 years [2], the recent emergence of positron emission tomography (PET) imaging of tau NFTs has allowed for in vivo characterization of this pathology and highlights the enduring importance of tau in the development and progression of AD [3]. Biomarker frameworks of AD, such as the amyloid-tau-neurodegeneration (AT[N]) framework, have capitalized on advancements in tau PET imaging to stage participants based on their biomarker profile and motivate research across the AD continuum using these staging efforts [4, 5].

Classification into AT(N) stages requires the designation of participants as Aβ, tau, and neurodegeneration positive or negative (A+/A−, T+/T−, [N+/N−]). Whereas cut-points for Aβ positivity have been well-validated and relatively consistently used [6–8], there has been considerable methodological variance in tau PET thresholding studies, resulting in discrepant cut-points with little consistency across studies, despite evidence suggesting strong reliability of tau PET [9]. Whereas Aβ is diffusely distributed and commonly quantified as a global cortical mean tracer uptake, tau accumulation shows a hierarchical pattern of spread [10]. Thus, the question of how to define tau positivity requires examination of both the quantity of tracer retention and its location, each of which can be defined in multiple ways. Importantly, there has not yet been a comprehensive characterization of these differing methods nor a compilation of the various tau PET cut-points currently in use. Such heterogeneity in sample characteristics and methods across studies directly influences the rates of tau positivity and, consequently, the proportions of each resultant AT(N) profile.

This study sought to clarify the impact of these methodological differences in two ways. First, we reviewed previous studies that have derived a cut-point for tau in AD and related phenotypes and comprehensively evaluated the thresholding methods used, as well as the influence of these methods on resultant cut-points. Second, to more systematically evaluate the unique influence of these methodological factors, we conducted an empirical follow-up study employing a systematic comparison of tau PET (^18^F-AV-1451, flortaucipir) thresholding methods that varies only one factor at a time (e.g., varying the analytic approach while holding the sample characteristics and preprocessing methods constant). This was conducted in order to assess the independent effects of sample ROIs, analytic approach, and comparison group/criterion variable (i.e., Aβ negative cognitively unimpaired [CU] or CU only) on tau cut-points and resultant tau positivity rates. We then evaluated the criterion validity of these varying methodological approaches to better inform future research using tau PET cut-points for AT(N) classifications or other AD staging paradigms as well as clinical treatment trial eligibility.

## Methods

### Review

**Fig. 1:**
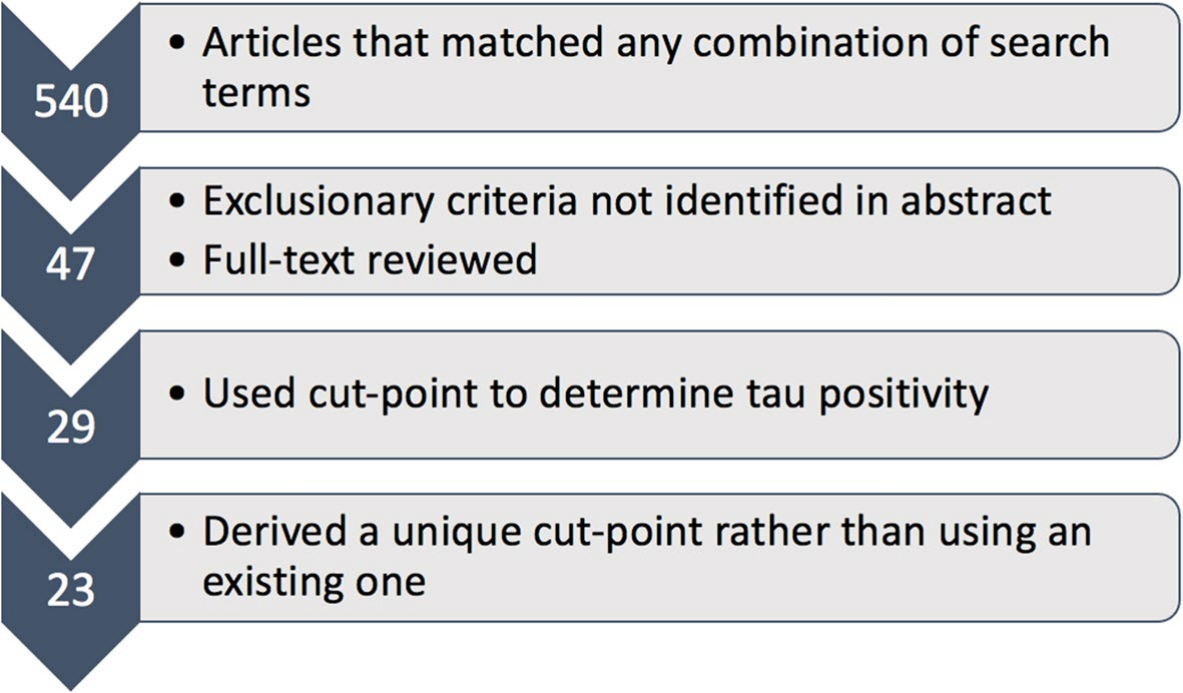
Flowchart depicting the process of selecting articles to include in this systematic review

A literature search conducted between January 1, 2020, and October 1, 2020, indexed PubMed/MEDLINE databases following PRISMA guidelines (http://www.prisma-statement.org/) to identify studies relevant to the current review. The following search terms were used: tau PET positivity, tau PET threshold, tau PET cutoff (including cut-off and cut off), tau PET cut-point (including cut-point and cut point). The search was then repeated with “tau PET” replaced by “flortaucipir” in all previous search terms. There was no restriction placed based on study year, and all studies from January 1, 1993, through October 1, 2020, were included. Exclusionary criteria were as follows: use of (1) a cut-point derived by another study rather than applying novel thresholding methods; (2) continuous tau PET measures with no cut-points; (3) a threshold derived for the purposes of non-binary classification (e.g., AD subtypes); (4) any tau PET tracer other than Flortaucipir; (5) cerebrospinal fluid rather than PET tau biomarkers; (6) non-AD neurological conditions as a focus (e.g., Parkinson’s disease); (7) animal models. All resulting articles were screened by checking the abstract for exclusionary criteria. If no exclusionary criteria were met based on the abstract or if it was unclear, the full text was reviewed to determine eligibility. Finally, any articles that passed full-text review were included and verified independently. See Fig. 1 for a flow diagram depicting this process. Quality ratings were conducted based on the Oxford Centre for Evidence-based Medicine (https://www.cebm.net/) by three independent raters with no discrepancies. Summary measures include descriptive statistics of tau PET cut-points based on different methods.

### Empirical examination of the varying factors influencing cut-points

In order to assess the reliability of different cut-point methods and their concurrent validity, we systematically varied the methodological approaches identified in this review within a sample of participants from the Alzheimer’s Disease Neuroimaging Initiative (ADNI). In this way, we controlled for the effects of sample size/characteristics and preprocessing methods that complicated comparisons across existing studies. Participants were included if they had tau PET (flortaucipir) and Aβ PET (florbetapir) imaging data within 12 months of one another and neuropsychological data, resulting in a sample of 453 participants.

Within the ADNI sample of 453 older adults, resultant cut-points were examined in a 2 × 2 × 2 design that varied methodological decision points. The 8 possible cut-points were derived from different combinations of region (entorhinal cortex or temporal meta-ROI), analytic approach (receiver operating characteristics (ROC) or 2 standard deviations above comparison group), and comparison group/criterion variable (Aβ negative CU or CU-only).

Full information on processing methods for ADNI Aβ PET (^18^F-AV-45, florbetapir) and tau PET (^18^F-AV-1451, flortaucipir) has been previously described elsewhere [11, 12]. Standardized uptake values (SUVs) were intensity normalized using the whole cerebellum (Aβ PET; [11, 13]) or inferior cerebellar gray (tau PET; [12, 14]) to create SUV ratios (SUVRs). Tau PET data were partial volume corrected using the geometric transfer method [15]. Aβ PET data closest in time to tau PET data were used, and all included Aβ PET data were collected within 12 months of the tau PET data. Only florbetapir and flortaucipir data were used to avoid confounding effects of different tracers.

Two regions of interest (ROIs) were examined for tau PET. A FreeSurfer-derived entorhinal cortex region (averaged across hemispheres) was used to approximate early Braak staging rather than an entorhinal cortex and hippocampus composite per extant recommendations given the high susceptibility of the hippocampus to partial volume effects [12]. A temporal meta-ROI was also examined using a composite of the following FreeSurfer-derived regions (averaged across hemispheres): amygdala, entorhinal cortex, fusiform gyrus, inferior temporal gyrus, and middle temporal gyrus.

Two analytic methods were examined. ROC analysis assessed the performance of predictive models based on a binary criterion variable [16]. In this case, tau PET SUVR (using either the temporal meta-ROI or entorhinal cortex) was used to predict classification as either Aβ positive/negative or cognitively unimpaired (CU)/impaired (CI). ROC curves represent classification accuracy as sensitivity (*y*-axis) versus 1 − specificity (*x*-axis). Optimal cut-points were defined using the Youden index (sensitivity + specificity − 1). The second analytic method defined cut-points for the temporal meta-ROI and entorhinal cortex as a tau PET SUVR that was 2 standard deviations above a comparison group (Aβ negative CU or CU-only).

Two separate criterion variables/comparison groups were used for each analytic approach. Aβ PET negativity, defined based on the existing cut-point of >1.11 [7, 11], along with cognitive diagnosis (CU) served as one criterion/comparison for defining tau PET cut-points. Cognitive diagnosis only (CU irrespective of Aβ status) served as the other criterion/comparison. Demographic and biomarker characteristics of the Aβ negative CU and CU-only group are reported in Table 1. Diagnosis of CU or mild cognitive impairment (MCI) was determined using Jak/Bondi comprehensive neuropsychological criteria [17] at the time of the tau PET scan. Participants were diagnosed with MCI if they (1) had two impaired scores in one cognitive domain or (2) had one impaired score across all three cognitive domains. Six individual neuropsychological measures across domains of memory (Auditory Verbal Learning Test delayed recall and recognition), language (Boston Naming Test/Multilingual Naming Test and animal fluency), and attention/executive function (Trail Making Test A and B) were included. Raw scores were then converted to *z*-scores based on predicted values from regression equations adjusting for age, sex, and education derived within a robust CU group (i.e., CU throughout their duration in ADNI) based on the entire ADNI sample. Diagnosis of dementia was made based on the following criteria utilized by ADNI (http://adni.loni.usc.edu/): (1) subjective memory complaint reported by the participant, study partner or clinician; (2) objective memory impairment defined by a score below education-adjusted cut-offs on Logical Memory Delayed Recall, Story A of the Wechsler Memory Scale—Revised; (3) score between 20 and 26 on the Mini-Mental State Examination; (4) score 0.5 or 1.0 on the Clinical Dementia Rating Scale; and (5) met NINCDS/ADRDA criteria [18] for probable Alzheimer’s disease. MCI and dementia groups were combined into one CI group.

**Table 1:** Descriptive characteristics of the A− CU and CU-only comparison groups used in the overall sample. For quantitative variables, values are presented as mean(standard deviation)

Group	Age	Sex	Education	Tau PET EC	Tau PET meta	Amyloid PET
A− CU	70.7(6.2)	54.6% female	16.6(2.6)	1.67(.3)	1.51(.1)	1.01(.1)
CU-only	71.4(6.4)	54.8% female	16.6(2.4)	1.75(.4)	1.57(.2)	1.13(.2)

*A−* amyloid negative, *CU* cognitively unimpaired, *EC* entorhinal cortex, *Meta* meta-temporal region of interest, *PET* positron emission tomography

For each of the derived 8 cut-points, tau positivity rates were determined. Concurrent validity of these 8 unique cut-points was assessed via three separate outcomes: (1) cerebrospinal fluid (CSF) phosphorylated tau (p-tau), (2) Mini-Mental State Examination (MMSE) total score, and (3) Logical Memory Story A Delayed Recall *z*-score (relative to robust CU group). Specifically, for each of the 8 cut-points, tau positive (T+) and tau negative (T−) participants were compared on these outcome variables, resulting in a Cohen’s *d* statistic that indicates the degree of discrepancy on these outcomes between T+ and T− participants for a given cut-point. Cohen’s *d* statistics were then used to qualitatively compare results across all 8 cut-points, with a larger Cohen’s *d* indicative of a higher concurrent validity for that cut-point. To avoid circularity, we split the 453 ADNI participants into a training dataset to derive cut-points (65%, *n* = 294) and test dataset to validate the cut-points (35%, *n* = 159) through non-replacement random sampling of the original dataset. Notably, the cut-points we derived are specifically applicable to the sample characteristics and preprocessing methods used within ADNI.

## Results

### Review

Based on the above search methods, 540 articles were screened and 47 had the full-text reviewed to determine eligibility. The inclusion criteria were then met by 23 studies (see Fig. 1 and Table 2) with a total of 6536 participants (note that this participant pool may not have been unique due to overlap in datasets utilized across the published studies). The quality of the evidence was equivalent across all studies included in this review with 23 cross-sectional studies each having a quality rating of 4 (on a scale of 1–5 with 1 being the highest quality) according to the Oxford Centre for Evidence-based Medicine (https://www.cebm.net/).

**Table 2:** Systematic review of 23 studies deriving tau PET cut-points

Study	Sample	Reference region	PVC	ROI(s)	Analytic approach	Cut-point
[19] Botha et al. (2018), *Brain*	24 CI OAs from MCSA and ADRC	Cerebellar crus gray	Not reported	Temporal meta-ROI (amygdala, EC, fusiform, parahippocampal, ITG, MTG)	ROC maximizing sensitivity/specificity between YAs and A+ CI OAs	1.33
[20] Cho et al. (2018), *Neurobiol Aging*	220 OAs (all dx) from memory clinic	Cerebellar cortex	Yes using region-based voxelwise method (and no)	25 individual ROIs	Mean + 2.5 SDs above CU with minimal cortical Abeta (<1.4) and EC tau (<1.2)	Not reported
[21] Dodich et al. (2020), *Eur J Nucl Med*	81 CI OAs from memory clinic	Cerebellar crus	Not reported	Meta-ROI (medial temporal lobe, lateral occipital, ITG)	ROC between A−/N− vs other	1.24
[22] Guo et al. (2020), *Alz Res Ther*	341 OAs (all dx) from ADNI	Inferior cerebellar cortex	Yes using GTM	(1) Temporal meta-ROI (amygdala, EC, fusiform, parahippocampal, ITG, MTG) (2) EC ROI	(A) ROC with Youden index between A− CU and A+ C (B) Mean +2 SDs above A− CU	(1A) 1.25 (2A) 1.21 (1B) 1.34 (2B) 1.31
[23] Jack et al. (2017), *Alz Dem*	49 YAs and 153 OAs (all dx) from MCSA and ADRC	Cerebellar crus gray median	“Most likely CSF” voxels were removed	Temporal meta-ROI (amygdala, EC, fusiform, parahippocampal, ITG, MTG)	(1) Maximize specificity (95%) based on YAs (2) Maximize sensitivity (10%) based on A+ CI OAs (3) ROC maximizing accuracy between YAs and A+ CI OAs (4) ROC maximizing accuracy between A− CU OAs and A+ CI OAs	(1) 1.19 (2) 1.21 (3) 1.21 (4) 1.32
[24] Jack et al. (2017), *Lancet Neuro*	435 OAs from MCSA (YAs not reported)	Cerebellar crus gray median	No	Temporal meta-ROI (amygdala, EC, fusiform, parahippocampal, ITG, MTG)	ROC maximizing accuracy between YAs and A+ CI OAs	1.23
[25] Jack et al. (2019), *JAMA*	480 OAs without dementia from MCSA (YAs not reported)	Cerebellar crus gray	No (and yes using two compartment method)	Temporal meta-ROI (amygdala, EC, fusiform, parahippocampal, ITG, MTG)	(1) ROC maximizing accuracy between YAs and A+ CI OAs (2) ROC maximizing accuracy between A− CU and A+ CI OAs	(1) 1.25 (2) 1.33
[26] Jang et al. (2019), *Eur J Nucl Med imaging*	60 OAs with SVCI from medical center and 49 OAs (all dx) from hospital	Cerebellar gray	Yes	(1) Braak V/VI (2) Braak III/IV (3) Braak I/II	CIDT (criterion variable unspecified); T+ considered III/IV or above	(1) 1.58 (2) 1.33 (3) 1.29
[27] Lowe et al. (2018), *Alz Dem: DADM*	112 YAs from MCSA and 576 OAs from MCSA and ADRC	Cerebellar Crus	No	Temporal meta-ROI (EC, parahippocampal, hippocampus); 26 individual ROIs also assessed	95% above A− YAs	Not reported
[28] Lowe et al. (2018), *Brain*	98 YAs, 601 A− CU OAs, 86 A+ CI OAs from MCSA and ADRC	Cerebellar crus gray	No (and yes using two compartment method)	47 individual ROIs	95% above A− YAs for each ROI	Not reported
[29] Lowe et al. (2019), *Neurology*	117 YAs; 579 CU OAs from MCSA	Cerebellar crus	No (and yes using GTM)	43 individual ROIs	95% percent above A− YAs	1.17 in EC
[30] Lowe et al. (2020), *Alz Dem*	26 OAs from MCSA and ADRC	Cerebellar Crus	No (and yes using GTM)	(1) Temporal meta-ROI (amygdala, EC, fusiform, parahippocampal, ITG, MTG) (2) EC ROI	ROC with Youden index between AD and non-AD spectrum pathology	* (1) 1.29 (2) 1.27
[14] Maass et al. (2017), *Neuroimage*	Sample 1: 12 YAs, 74 CU OAs from BACS, and 61 CI OAs from ADRC Sample 2: 42 CU and 28 CI OAs from ADNI	Inferior cerebellar gay	(1) Yes using GTM (2) No	(A) Temporal meta-ROI (B) ITG (Also examined whole brain, AD-vulnerable mask, and Braak stage composite regions)	ROC with Youden index between A− CU OAs and A+ CI OAs) (Also used CIDT with age/diagnostic group as input variable for Braak composite staging)	(1A) 1.47 (BACS/UCSF) and 1.34 (ADNI) (1B) 1.27 (BACS/UCSF) and 1.20 (ADNI) (2A) 1.46 (BACS/UCSF) and 1.40 (ADNI) (2B) 1.30 (BACS/UCSF) and 1.23 (ADNI) (other cut-points omitted from table but included in results section)
[31] Mattsson-Carlgren et al. (2020), *Sci Adv*	131 OAs (all dx) from BioFINDER	Inferior cerebellar gray	No (and yes using GTM in EC)	(1) EC (2) ITG (3) Braak V/VI	Mean +2 SDs above independent A− CU group	(1) 1.39 (2) 1.31 (3) 1.28
[32] Meyer et al. (2020), *JAMA Neuro*	322 OAs (all dx) from ADNI	Inferior cerebellar gray	No (and yes using GTM)	Temporal meta-ROI (amygdala, EC, fusiform, parahippocampal, ITG, MTG)	ROC with maximum percentage correct classification between A− CU OAs and A+ CI OAs	* 1.37
[33] Mishra et al. (2017), *Neuroimage*	97 OAs (all dx) from ADRC	(1) Whole cerebellum (2) Cerebellar cortex	Yes using RSF	Meta-ROI (EC, lateral occipital, ITG, amygdala)	SKM method to cluster into high and low tau groups; midpoint taken	(1) 1.25 (2) 1.22
[34] Ossenkoppele et al. (2018), *JAMA*	719 OAs (all dx) from ADRC, BioFINDER, and memory clinic	Inferior cerebellar gray	No (and yes using GTM)	5 ROIs examined: EC, ITG, temporal meta-ROI, temporoparietal cortex, Braak V/VI	(1) Mean +2 SDs above CU (2) ROC Youden index between controls and AD	* For temporal meta-ROI: (1) 1.34 (2) 1.27 (other cut-points omitted from table but included in results section)
[35] Rafii et al. (2017), *JAD*	9 adults with Down syndrome from DSBI	(1) Cerebellar cortex (bottom slice removed and edges eroded) (2) Subcortical WM	Yes using Muller-Gartner method	Braak I-VI average	Quantitative discrimination of A− vs A+	(1) 1.2 (2) 1.05
[36] Schöll et al. (2016), *Neuron*	5 YAs and 33 CU OAs from BACS; 15 CI OAs from ADRC	Cerebellar gray	Yes using GTM	(1) Braak V/VI (2) Braak III/IV (3) Braak I/II	CIDT using age/diagnostic group as input variable	(1) 2.79 (2) 1.73 (3) 1.40
[37] Schwarz et al. (2016), *Brain*	14 YAs and 173 OAs (all dx) from a clinical study	Cerebellar crus	Not reported	7 ROIs corresponding to Braak histological stages	Mean +2.5 SDs above YAs	Ranged from 1.22 (STG) to 1.36 (fusiform)
[38] Schwarz et al. (2018), *Alz Dem*	14 YA, 21 OAs (all dx) for test-retest, and 98 OAs (all dx) from ADNI	Cerebellar gray	Not reported	(1) Meta-ROI (hippocampus, TEC, fusiform, MTG, STG, extrastriate, striate) (2) Simplified meta-ROI (medial temporal, lateral temporal, STG, primary visual cortex) (3) Lobar (average temporal, parietal, and frontal lobes) (ROIs gray matter masked)	(1) Mean +2.5 SDs above T− YAs for each ROI within meta-ROI; T+ includes those above threshold in hippocampus, TEC, fusiform, MTG, and extrastriate (2) Mean +3 SDs above T− YAs to obtain same average as (1); T+ includes those above threshold in MTL and lateral temporal (3) Mean +3 SDs above T− YAs to obtain same average as (1); T+ includes those above threshold in temporal lobe	* 1.28
[39] Wang et al. (2016), *JAMA Neuro*	59 OAs (all dx) from ADRC	Cerebellar cortex	No (and yes using linear regression)	(1) Hippocampus (2) Meta-ROI (medial temporal, ITG, lateral temporal, inferior parietal, PCC, precuneus, SPL)	(A) ROC with Youden index between A− CU and A+ AD (B) ROC with Youden index between A+ CU and A+ AD	* (1A) 1.36 (2A) 1.19 (1B) 1.36 (2B) 1.33
[40] Weigand et al. (2020), *Brain Comms*	523 OAs (all dx) from ADNI	Inferior cerebellar gray	Yes using GTM	(1) Braak V/VI (2) Braak III/IV (3) Braak I/II	CIDT with MMSE as input variable	(1) 1.96 (2) 1.51 (3) 1.18

*A* amyloid, *ADNI* Alzheimer’s Disease Neuroimaging Initiative, *ADRC* Alzheimer’s Disease Research Center, *BACS* Berkeley Aging Cohort Study, *CIDT* conditional inference decision tree, *CU* cognitively unimpaired, *CI* cognitively impaired, *DSBI* Down Syndrome Biomarker Initiative, *Dx* diagnoses, *EC* entorhinal cortex, *GTM* geometric transfer matrix, *ITG* inferior temporal gyrus, *MCSA* Mayo Clinic Study of Aging, *MTG* middle temporal gyrus, *N* neurodegeneration, *OAs* older adults, *PCC* posterior cingulate cortex, *ROC* receiver operating characteristics, *ROI* region of interest, *SD* standard deviation, *SKM* sparse k-means, *SPL* superior parietal lobe, *STG* superior temporal gyrus, *SVCI* subcortical vascular cognitive impairment, *T* tau, *TEC* transentorhinal cortex, *UCSF* University of California San Francisco, *YAs* younger adults

*Additional cut-points are reported in the supplementary material of these studies

#### Cut-points

A total of 82 cut-points were reported in the main text across the 23 studies (note additional cut-points reported as supplementary material were excluded for the sake of parsimony, but such studies are marked in Table 2). The cut-points derived in the included studies ranged from 1.13 to 2.79 (the next highest value was considerably lower at 1.96). The mean value across all cut-points was 1.33 with a standard deviation (SD) of 0.21, and a median of 1.29. With the outlier value of 2.79 removed, the mean was 1.31 with a SD of 0.14, with a median of 1.28. Given that this variability may be due to several methodological differences, we explored this further below.

#### Sample characteristics

Inclusion criteria yielded a sample of 6115 cognitively unimpaired and cognitively impaired older adults, as well as 421 younger adults who served as reference groups. In addition to older adults who were cognitively unimpaired or diagnosed with mild cognitive impairment, many studies included individuals diagnosed with dementia. While the majority of studies recruited participants with probable AD, other dementia syndromes included AD variants (i.e., dysexecutive AD, posterior cortical atrophy; [14, 36]), hippocampal sclerosis [19], subcortical vascular cognitive impairment [26], and non-AD neurodegenerative disorders (e.g., Lewy body dementia, primary progressive aphasia; [34]). The current review also included one study investigating AD in individuals with Down syndrome [35].

Several included studies recruited participants from research initiatives such as the Mayo Clinic Study of Aging (MCSA; *n* = 8), Alzheimer’s Disease Neuroimaging Initiative (ADNI; *n* = 5), Berkeley Aging Cohort Study (BACS; *n* = 2), BioFINDER (*n* = 2), and Down Syndrome Biomarker Initiative (DBSI; *n* = 1). Other sources included local Alzheimer’s disease research centers (*n* = 10), memory/medical clinics (*n* = 4), or a clinical study (*n* = 1). Note that several studies recruited from multiple sources to increase the generalizability of the findings, and therefore the above total (33) exceeds the number of included studies (23). See Table 2 for further details.

#### Preprocessing methods

Differences were observed in preprocessing methods including reference region and use of partial volume correction (PVC), which may contribute to inter-study variability in cut-points. Twenty-one studies used a cerebellar region, with the cerebellar gray (*n* = 6) and inferior cerebellar gray (*n* = 6) as the most commonly used reference regions to calculate SUVRs. Other regions included the cerebellar crus (*n* = 5), cerebellar crus gray (*n* = 3), and cerebellar crus gray median (*n* = 2; note that all other reference regions were calculated using mean as the measure of central tendency). The whole cerebellum and subcortical white matter were also used.

**Fig. 2:**
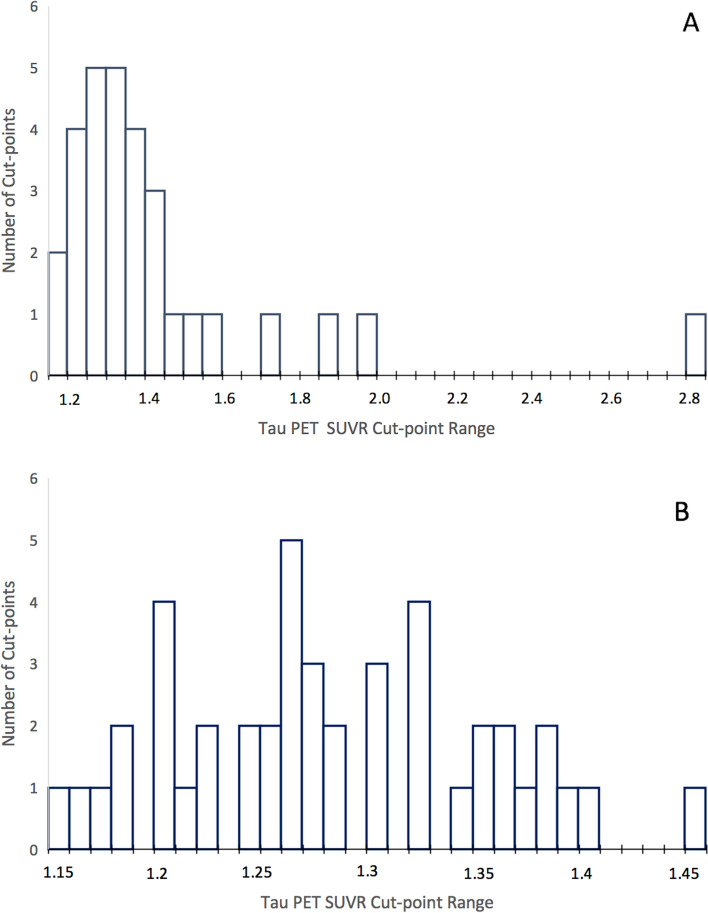
Distribution of tau PET SUVR cut-points in methods with (**A**) or without (**B**) partial volume correction (PVC). PET = positron emission tomography. SUVR = standardized uptake value ratio

Use of PVC was indicated by 19/23 studies, with 6 only using PVC, 3 not using any PVC (note that one of these studies did remove “most likely CSF” voxels), 4 not reporting either way, and 10 directly comparing PVC and non-PVC approaches. On average, use of PVC resulted in a higher cut-point (PVC mean[SD] = 1.41[.32], or 1.37[.20] with outlier of 2.79 removed; non-PVC = 1.29[.07]). See Fig. 2 for a distribution of cut-points based on use of PVC. See Table 2 for further details.

#### Regions of interest

**Fig. 3:**
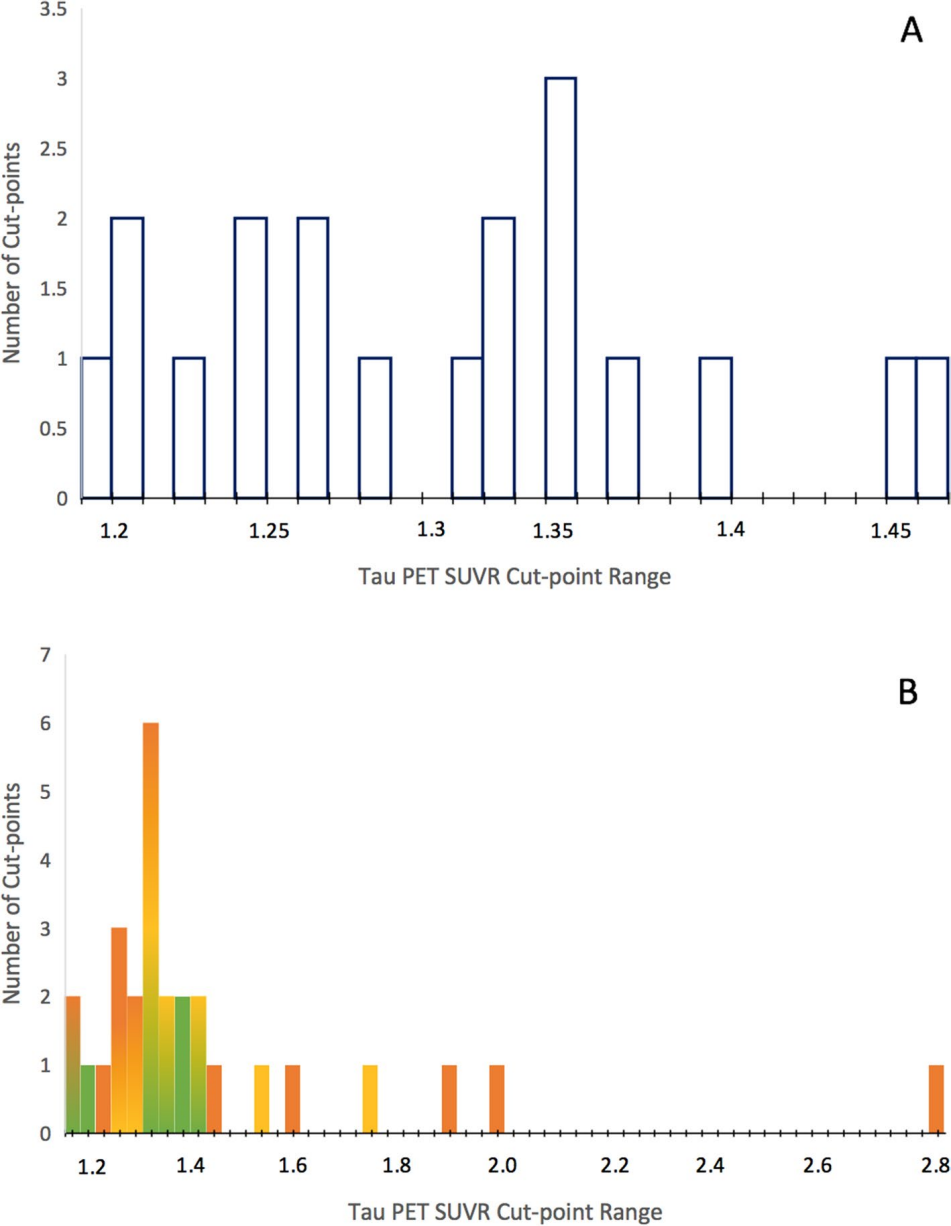
Distribution of tau PET SUVR cut-points in methods using a temporal meta-ROI (**A**) or Braak stage ROIs (**B**). PET = positron emission tomography. SUVR = standardized uptake value ratio. Green depicts Braak stage I/II; yellow depicts Braak stage III/IV; orange depicts Braak stage V/IV; note that colors may overlap

Several regions of interest (ROIs) were used to derive cut-points in the reviewed studies, including individual and composite ROIs. A temporal meta-ROI that was first introduced by Jack et al. [23, 24] consisting of various medial and lateral temporal regions was used by the largest proportion of studies (10/25; cut-point mean[sd] = 1.31[.08]). Similarly, 4 studies thresholded on a larger meta-ROI that extended to occipital or parietal regions, with one of these studies additionally assessing lobar ROIs. Braak stage composites were used by 8 studies, typically including stages I/II (cut-point mean[SD] = 1.29[.09], III/IV (1.38[.16]), and V/VI (1.52[.48], or 1.41[.28] with outlier removed). Additionally, 5 studies selected individual ROIs such as the entorhinal or inferior temporal cortex, and 4 other studies assessed a large number of ROIs throughout the brain. See Table 2 for further details and Fig. 3 for distributions of cut-points using a temporal meta-ROI or Braak stage composites. Notably, these statistics are averaged across studies using or not using PVC, which will also contribute to variability.

#### Thresholding analytic procedures

Perhaps the most heterogeneity in tau PET thresholding methods was observed in the type of analysis used, with only a few of the 23 studies employing the same particular analytic approach. There were, however, clusters of similar statistical methods employed across studies that varied in aspects such as criterion variable or groups assessed. The most frequently observed of these analytic “clusters” was use of receiver operating characteristics (ROC) curves discriminating between two groups to identify the optimal cut-point for differentiating tau positive and negative individuals, which was used in 11 studies. This method varied, however, in the criterion variable (i.e., groups) predicted by tau PET SUVRs, with various combinations of younger adults, Aβ negative (A−) or Aβ positive (A+) older adults, and CU or cognitively impaired (CI) older adults. The second most frequently observed analytic “cluster” was identifying a threshold (e.g., 2 standard deviations) above the average tau PET SUVR from a reference group at which the cut-point would be defined (*n* = 9). With this method, there was substantial variability across studies in the reference group used, including younger adults, A− CU older adults, and all CU older adults. See Table 2 for further details.

### Empirical examination of the varying factors influencing cut-points

The cut-point values and resultant tau positivity rates for each of the 8 cut-points derived in the ADNI test sample (using PVC data) are reported in Table 3. The average cut-point when using the “early” (i.e., entorhinal) ROI was 2.19, whereas the average cut-point when using the “late” (i.e., meta-temporal) ROI was 1.80. This discrepancy is unsurprising given that, within the same sample, we would expect higher tau deposition within the “early” ROI relative to the “late” ROI as delineated by the spatiotemporal progression of tau based on Braak staging, necessitating a higher threshold for the “early” ROI. This cut-point for each ROI, however, resulted in similar tau positivity rates, on average, with 27.8% tau positivity for the “early” ROI and 24.4% tau positivity for the “late” ROI, indicating no appreciable effect of ROI-independent of analytic method and comparison group/criterion variable on tau positivity rates.

**Table 3:** Derivation of tau PET cut-points, resultant tau positivity rates, and Cohen’s *d* statistics discriminating tau positivity/negativity for 8 unique thresholding methods

Method	Cut-point	T+ rate	CSF p-tau Cohen’s ***d***	MMSE Cohen’s ***d***	Logical memory Cohen’s ***d***
Entorhinal/ROC/A− CU	1.97	32.5%	− .80 (95% CI [− 1.15, −.80])	1.14 (95% CI [.87, 1.40])	1.17 (95% CI [.90, 1.44])
Entorhinal/ROC/CU	1.94	37.6%	− 1.00 (95% CI [− 1.35, −.65])	1.12 (95% CI [.85, 1.38])	1.12 (95% CI [.86, 1.38])
Entorhinal/2SD/A− CU	2.39	21.5%	−.96 (95% CI [−.1.39, −.52])	**1.65 (95% CI [1.32, .1.97])**	**1.65 (95% CI [1.33, 1.97])**
Entorhinal/2SD/CU	2.45	19.7%	− 1.06 (95% CI [− 1.50, −.61])	1.55 (95% CI [1.22, 1.87])	1.54 (95% CI [1.21, 1.86])
Meta-ROI/ROC/A− CU	1.64	38.7%	− 1.18 (95% CI [− 1.54, −.82])	1.13 (95% CI [.86, 1.39])	.90 (95% CI [.65, 1.16])
Meta-ROI/ROC/CU	1.68	32.8%	− 1.26 (95% CI [− 1.63, −.89])	1.25 (95% CI [.97, 1.52])	.99 (95% CI [.72, 1.25])
Meta-ROI/2SD/A−CU	1.78	22.2%	−**.1.81 (95% CI [**−**.2.26,** −**.1.35])**	**1.67 (95% CI [1,34, 1.98])**	1.53 (95% CI [1.21, 1.84])
Meta-ROI/2SD/CU	2.08	13.9%	− 1.27 (95% CI [− 1.80, −.74]	**1.67 (95% CI [1.30, 2.04])**	1.42 (95% CI [1.06, 1.79])

*A−* amyloid negative, *CSF* cerebrospinal fluid, *CU* cognitively unimpaired, *MMSE* Mini-Mental State Examination, *ROC* receiver operating characteristics, *SD* standard deviation, *T* tau. The largest effect sizes for each outcome variable are presented in bold

**Fig. 4:**
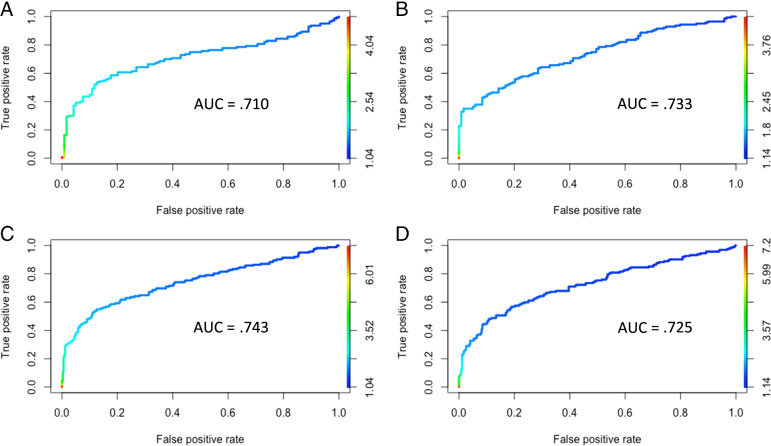
Receiver operating characteristics (ROC) curves used to derive cut-points for **A** entorhinal cortex with Aβ negative CU as the criterion, **B** temporal meta-ROI with Aβ negative CU as the criterion, **C** entorhinal cortex with CU-only as the criterion, and **D** temporal meta-ROI with CU-only as the criterion. AUC = area under the curve. Aβ = amyloid-beta

The average cut-point when using ROC analysis was 1.81, whereas the average cut-point when positivity is defined as 2 standard deviations above a comparison group was 2.18. On average, there was a 32.9% tau positivity rate when using ROC analysis and a 19.3% tau positivity rate with an analytic method of 2 standard deviations above a comparison group. Thus, use of the 2 standard deviation method resulted in a more conservative cut-point and resultant tau positivity estimate independent of ROI and comparison group/criterion variable. ROC figures with area under the curve (AUC) values for each ROI and criterion variable are presented in Fig. 4.

The average cut-point when using an Aβ negative CU comparison group/criterion variable was 1.95, whereas the average cut-point when using a CU-only comparison group/criterion variable (regardless Aβ status) was 2.06. On average, there was a 28.7% tau positivity rate when defining groups based on Aβ negativity and cognitive diagnosis and a 26% tau positivity rate when defining groups based on cognitive diagnosis only. Thus, the addition of Aβ status to cognitive diagnosis as the comparison group/criterion variable does not, on average, confer a notable effect on positivity rates independent of ROI and analytic methods.

**Fig. 5:**
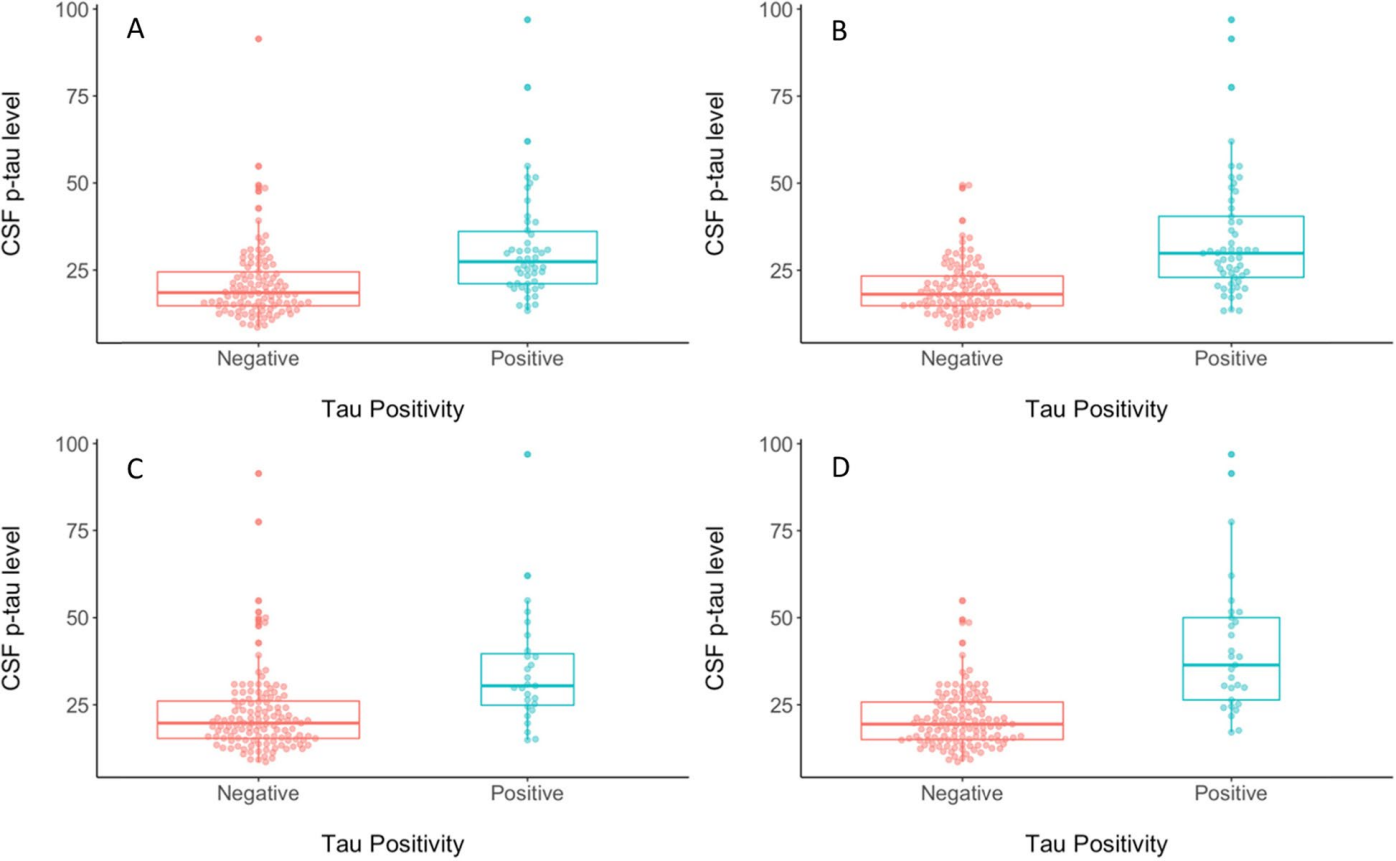
Beeswarm plots depicting differences between tau PET negative (red) and tau PET positive (blue) groups on CSF p-tau level when tau PET positivity is determined using a cut-point derived using **A** ROC with A− CU as the criterion in the EC, **B** ROC using A− CU as the criterion in the meta-ROI, **C** 2 SD above A− CU comparison group in the EC, or **D** 2 SD above A− CU comparison group in the meta-ROI. A− = amyloid negative. CSF = cerebrospinal fluid. CU = cognitively unimpaired. EC = entorhinal cortex. ROC = receiver operating characteristics. ROI = region of interest. SD = standard deviation

**Fig. 6:**
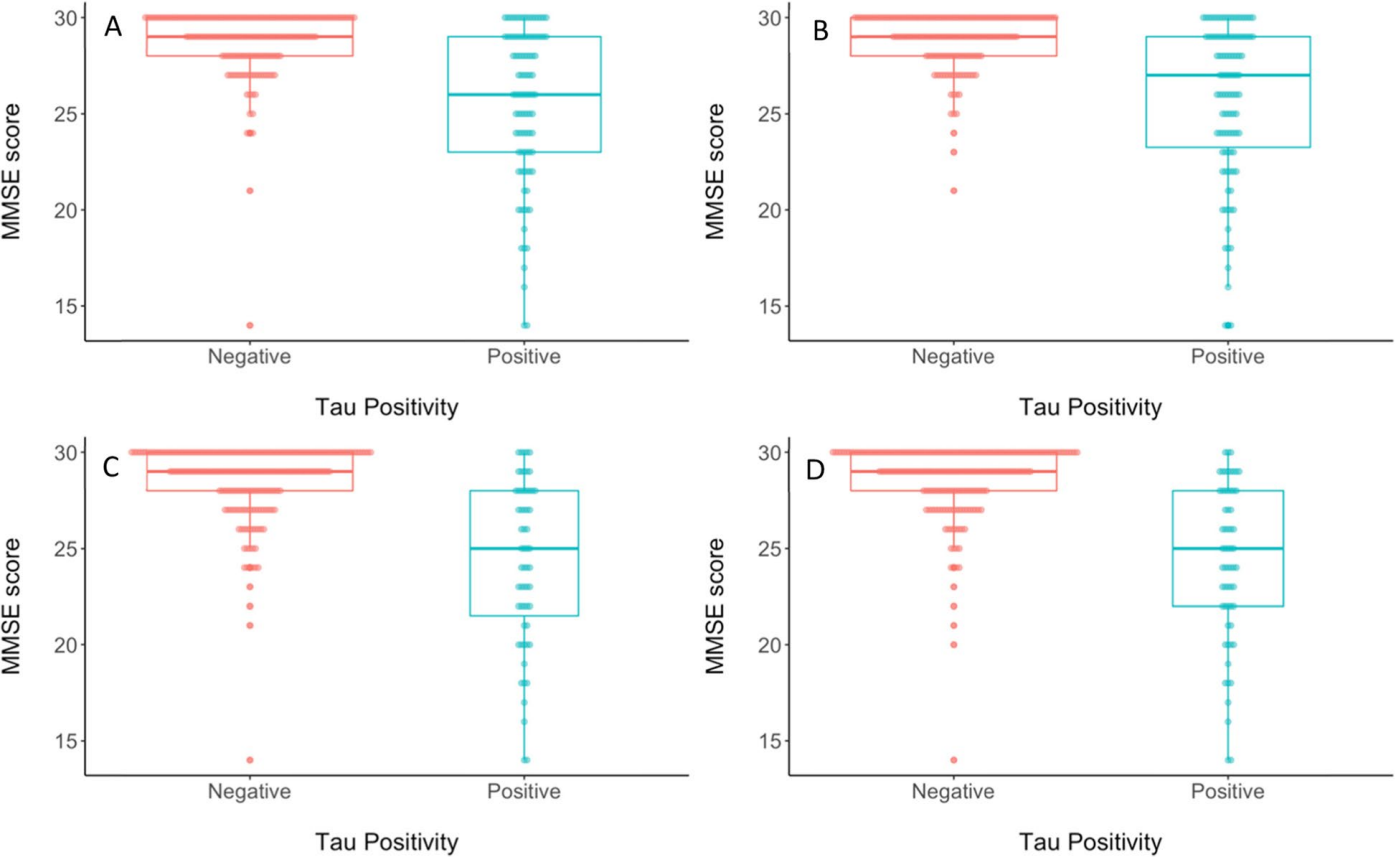
Beeswarm plots depicting differences between tau PET negative (red) and tau PET positive (blue) groups on MMSE score when tau PET positivity is determined using a cut-point derived using **A** ROC with A− CU as the criterion in the EC, **B** ROC using A− CU as the criterion in the meta-ROI, **C** 2 SD above A− CU comparison group in the EC, or **D** 2 SD above A− CU comparison group in the meta-ROI. A− = amyloid negative. CU = cognitively unimpaired. EC = entorhinal cortex. MMSE = Mini-Mental State Exam. ROC = receiver operating characteristics. ROI = region of interest. SD = standard deviation

**Fig. 7:**
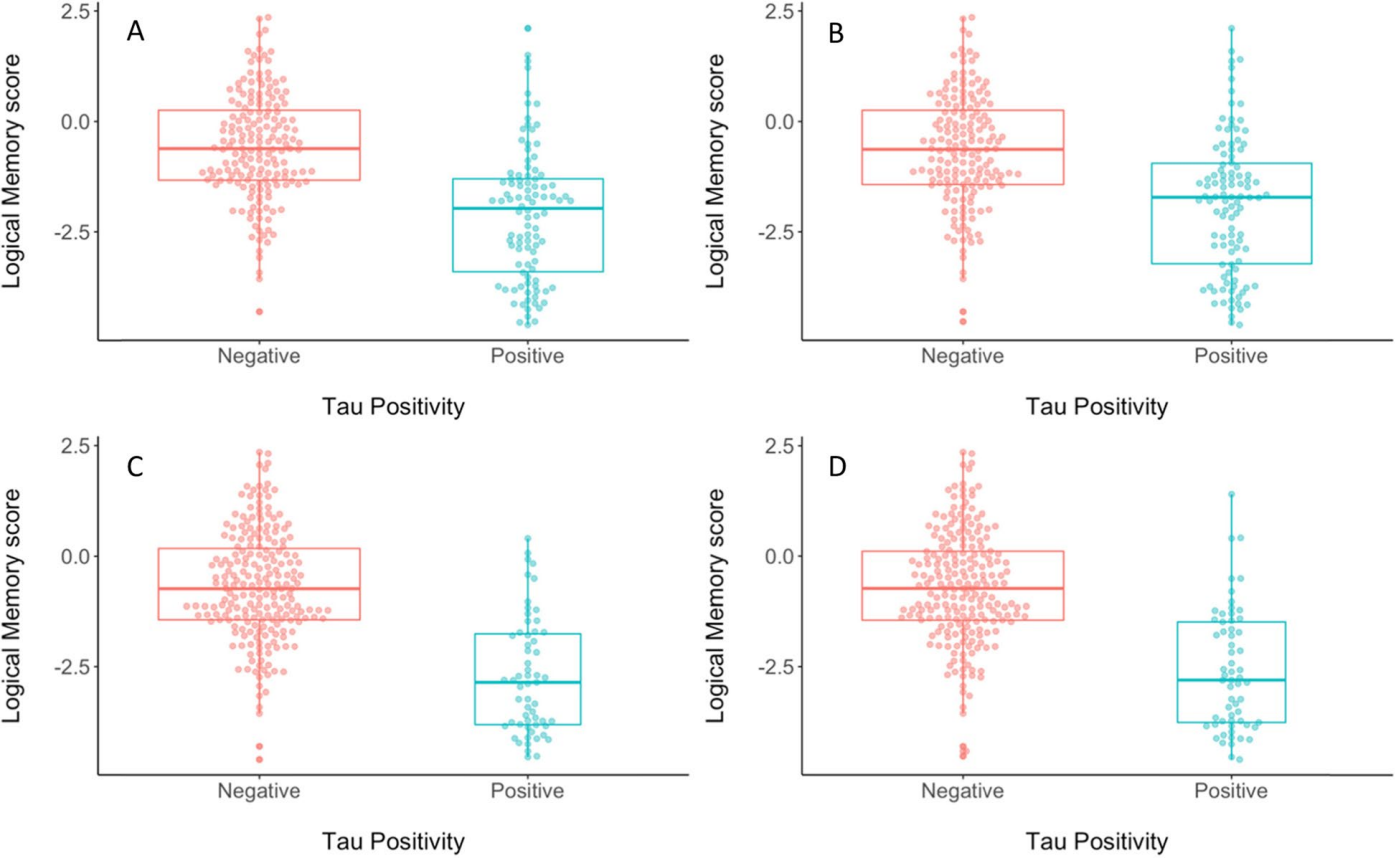
Beeswarm plots depicting differences between tau PET negative (red) and tau PET positive (blue) groups on logical memory delayed recall *z*-score when tau PET positivity is determined using a cut-point derived using **A** ROC with A− CU as the criterion in the EC, **B** ROC using A− CU as the criterion in the meta-ROI, **C** 2 SD above A− CU comparison group in the EC, or **D** 2 SD above A− CU comparison group in the meta-ROI. A− = amyloid negative. CU = cognitively unimpaired. EC = entorhinal cortex. ROC = receiver operating characteristics. ROI = region of interest. SD = standard deviation

Each of the 8 unique methods for deriving cut-points were then assessed for concurrent validity by evaluating differences between tau positive (T+) and tau negative (T-) participants from the ADNI test subsample for each cut-point on CSF p-tau, MMSE score, and logical memory delayed recall (see Table 3). When considering differences in CSF p-tau levels, the largest discrepancy between T+ and T− groups, as measured by Cohen’s *d*, was observed for the cut-point that was derived using 2 standard deviations above the mean of the Aβ negative CU group in the meta-temporal ROI (Cohen’s *d* = − 1.81; see Fig. 5). When considering differences in MMSE score, the largest discrepancy between T+ and T− groups, as measured by Cohen’s *d*, was observed for the cut-points that were derived based on 2 standard deviations above the Aβ negative CU or CU-only groups regardless of ROI (Cohen’s *d* ranged from 1.55 to 1.67 for 2 SD method vs. 1.12 to 1.25 for ROC method; see Fig. 6). When considering differences in Logical Memory delayed recall, the largest discrepancy between T+ and T− groups, as measured by Cohen’s *d*, was observed for the cut-point that was derived based on 2 standard deviations above the Aβ negative CU in the entorhinal cortex, although in general Cohen’s *d* was higher when using the 2 standard deviation method regardless of comparison group or ROI (ranged from 1.42 to 1.65 for 2 SD method vs. .90 to 1.17 for ROC method; see Fig. 7).

## Discussion

This review and empirical examination of cut-points based on varying threshold derivation methods and analyses revealed notable variability in tau PET SUVR cut-points across published studies to date, ranging from 1.13 to 2.79, and highlighted some of the possible sources and differences in methods to which this variability may be attributed. A discussion of the various decision points in thresholding methods is detailed below, including sample composition, preprocessing steps (i.e., reference region, PVC), selection of ROIs, and statistical approaches.

Firstly, samples differed in demographic and clinical characteristics. The choice of the reference group was thought to affect cut-points since entorhinal tau is common in cognitively normal older adults, which results in higher cut-points for abnormal tau relative to use of a younger control group. Similarly, age and clinical syndrome of the AD patients have a considerable effect on the cut-points. While higher tau tracer uptake in neocortical regions and thus higher cut-points are associated with younger age-of-onset and non-amnestic clinical presentations of AD patients, greater medial temporal lobe vulnerability is seen in older patients with amnestic symptoms such as in the ADNI sample (e.g., [14]).

As shown in the results, differences in preprocessing methods may also contribute to cut-point variability across studies. Interestingly, two studies directly compared results using different reference regions. Mishra et al. [33] compared both the whole cerebellum and the cerebellar gray as reference regions, with a marginally higher cut-point derived from use of the whole cerebellum reference region (SUVR = 1.25) relative to the cerebellar gray (SUVR = 1.22). Additionally, Rafii et al. [35] assessed multiple reference regions that resulted in a cut-point of 1.20 using a cerebellar gray reference (with the bottom slice removed and edges eroded) and a notably lower cut-point of 1.05 when using a subcortical white matter reference. This was the only study in this corpus of studies for review that included a non-cerebellar reference region, so further comparisons of cerebellar vs. white matter and their influences on cut-point values are not available at this time. More research is needed directly comparing cut-points derived from SUVRs based on differing reference regions to determine the magnitude of this effect on the resultant cut-points. Based on the data available from this single study, it appears that using subcortical white matter resulted in a substantially lower cut-point relative to the commonly used cerebellar gray reference region, possibly due to differences in off-target binding in these regions with the Flortaucipir tracer.

Use of PVC also varied considerably across studies, and many studies directly compared cut-points based on data that had or had not undergone PVC. For example, one study [32] found a much higher cut-point when using PVC (SUVR = 1.79) relative to non-PVC (SUVR = 1.37). This discrepancy is likely explained by the higher atrophy that is associated with higher tau pathology, which leads to partial voluming and thus underestimation of tau PET signal in subjects with advanced tau pathology. The indecision across (and within) studies on whether to use PVC indicates the importance of determining a standardized preprocessing approach to tau PET thresholding in order to reduce cut-point variability and facilitate interpretation of positivity rates.

In addition to the above differences in methodology, what varied most considerably across studies was (1) the region(s) on which cut-points were based and (2) the analytic choices used to derive cut-points. Thresholding procedures for tau PET necessitate the selection of an ROI that is susceptible to tau pathology. A single brain region may be used for a sensitive and localized analysis, or a composite of several regions may be used to cover multiple key regions, likely with increased reliability. As noted in Table 2, the majority of studies used a composite of some type, whether combining AD-vulnerable regions or recapitulating Braak stages [10]. A smaller number of studies utilized single ROIs in the medial temporal lobe implicated early in the AD pathologic process; although this approach offers increased sensitivity and may be appropriate in very early stages in which tau is confined to the entorhinal cortex [10], it may not capture the more widespread distribution of tau in later disease stages. Further, given that medial temporal structures are often subject to partial volume effects due to proximity to the choroid plexus [41], use of these regions as isolated ROIs may not be as advisable. Alternatively, use of a temporal meta-ROI may provide increased reliability of the estimate with only a marginal decrease in the sensitivity needed to detect early-stage tau pathology.

The type of statistical analysis used also varied significantly, although most approaches were based on discrimination against a comparison group (e.g., ROC with A− and A+ older adults, 2.5 SDs above mean value for young adults). In examination of the analytic procedures employed across studies, a theme emerged: studies derived a tau threshold that was either contingent on Aβ in some manner or was independent of Aβ. Those with Aβ-contingent methods were used in more than half of the included studies and are based on the assumption that A− controls are not on the AD pathway [5]. Alternatively, statistical approaches not contingent on Aβ instead used a comparison group of younger adults or older adults irrespective of Aβ status, or used cognitive status as a criterion variable. Notably, the contingency on Aβ used in some studies, based on the assumption that Aβ negative controls are not on the AD pathway, may influence resultant tau PET cut-points and was examined in our empirical follow-up.

Variability in tau PET cut-points inevitably leads to variability in tau positivity rates, which impacts subsequent staging efforts based on biomarker positivities and possible inclusion in clinical treatment trials, as well as increasing estimation uncertainty which hinders reproducibility in AD biomarker research. Using the different methodological approaches presented above yielded remarkably different cut-points, making it difficult to evaluate the variable utility of any single method. This served as the basis for our empirical investigation of cut-point methods (i.e., ROI, analytic method, and comparison group) to examine how different methodological decision points in a sample standardized on size, characteristics, and preprocessing techniques would influence cut-point values, resultant positivity rates, and concurrent validity with other biomarker and cognitive outcomes. Notably, the cut-points we derived are specifically applicable to the sample characteristics and preprocessing methods used within ADNI.

There was no appreciable difference in tau positivity rates, on average, based on ROI or comparison group/criterion variable (i.e., Aβ negative CU or CU-only). However, the analytic method used did yield a notable difference in cut-points and positivity rates such that use of 2 standard deviations above either comparison group, within either ROI, yielded higher cut-point values and lower tau positivity rates. It should be noted that, on average, the cut-points derived in this study were higher than many reported in the literature. This may be due to factors such as the use of PVC data in our analyses, for which the systematic review also revealed a higher cut-point average relative to non-PVC, as well as our use of Jak/Bondi criteria for diagnosis of cognitive impairment, which is less susceptible to false positive diagnostic errors [42].

Interestingly, the 2 standard deviation analytic method had the best predictive validity when examining the magnitude of discrepancies in MMSE score and memory recall between T+ and T− groups (as measured by Cohen’s *d* effect sizes), with a marginal increased effect in the entorhinal cortex relative to the meta-temporal ROI for memory recall. This slight increase in prediction of memory scores when deriving tau positivity thresholds based on the entorhinal cortex aligns with the high sensitivity of memory recall relative to a global screening measure, as well as the specificity of memory to the entorhinal region. When examining effect sizes for CSF p-tau, the 2 standard deviation method again generally outperformed the ROC method, although in this case the largest discrepancy was specific to the Aβ negative CU comparison group within the meta-temporal ROI.

These results are critical to improve our understanding how these various methodological decisions and different choices influence the derivation of tau PET cut-points and resultant positivity rates, and which approaches may be most appropriate to include in a standardized approach to tau PET biomarker thresholding based on their concurrent validity with CSF p-tau levels, MMSE score, and memory recall. In general, the 2 standard deviation analytic approach yielded higher tau thresholds and thus more conservative tau positivity estimates which was related to increased predictive validity regarding cognition and CSF p-tau over the ROC analytic approach. That said, ROC analytic approaches have utility in sensitivity/specificity metrics for differentiating groups, which can offer important information in threshold selection.

Importantly, the specific research question under investigation should determine the methodological approach used, which may necessitate use of certain ROIs, analyses, and/or comparison variables. For example, use of an entorhinal ROI may increase sensitivity to detect tau positive individuals early in their trajectory, whereas use of a meta-temporal ROI may prioritize specificity to ensure that individuals categorized as tau positive are indeed on an Alzheimer’s trajectory. It is worth noting, however, that although there was a slightly lower (i.e., more conservative) tau positivity rate when using the meta-temporal ROI (24.4%), this did not appreciably differ from the tau positivity rate using the entorhinal ROI (27.8%). Alternatively, our results indicated that inclusion of Aβ-negativity resulted only in a marginal difference in effect size for predicting cognitive performance compared to use of diagnosis (CU) alone (as the comparison variable for deriving cutpoints). Thus, if the goal of defining tau positivity groups is to predict cognitive performance and Aβ data are not available, use of cognitive diagnosis only as the comparison group (particularly in the entorhinal cortex) may offer the best predictive utility. Additionally, it should be noted that binarization of tau PET values may not be the most ideal method for all research questions. Retaining the original quantitative units, or else using a stepwise staging method consistent with Braak staging, may improve prediction of outcomes including AD progression. However, binarization of tau PET values in positive and negative groups has utility in certain situations, such as inclusion in clinical trials.

Rather than offering specific recommendations as to which thresholding methods to use, we urge researchers to carefully consider the ultimate goals of their use of tau positivity groups when determining a thresholding method for their specific study given the significant heterogeneity that can result from different methods. Indeed, one single standardized thresholding approach may not exist. A better understanding of thresholding procedures, and intention when selecting an approach for a given research question, may increase reliability and reproducibility of our research and help advance our understanding of biomarker dynamics across the AD continuum.

### Limitations

The primary limitation of this review is the difficulty in comparing how a specific method influences cut-point values due to the highly discrepant procedures and its various combinations across studies. For example, comparing analytic procedures either contingent or not contingent on Aβ status proved difficult since any given study with these groups may have used different reference regions, PVC applications, regions of interest, or even the method by which the determination of Aβ positivity was achieved. Such a comparison is important given the notion that tau may operate earlier than and independent of Aβ [43, 44]. Although we sought to address this in our empirical follow-up, in which a consistent sample with the same preprocessing methods was used to compare ROIs, analytic methods, and comparison group/criterion variable, our study did not directly investigate these other methodological variations that certainly contribute to variability in cut-points and resultant positivity rates. For example, use of PVC, intensity normalization method, and acquisition parameters such as spatial resolution and timing of acquisition likely also influences outcomes [45–48]. These were not investigated in our study in order for parsimony in comparisons, but their effect on cut-points should be systematically assessed in future studies given prior research demonstrating the importance of these factors [49–51]. Additionally, non-flortaucipir tau PET tracers were not investigated; comparison of these tracers and use of a centiloid-based analytic approach may improve standardization across studies with different tau PET tracers. Finally, although neuropathologic data were not available in our study for tau PET cut point determination or validation, use of these data as the gold standard to determine the presence of AD-related neurofibrillary tangles should be investigated in future research.

Another limitation of the review is indicated by the quality of evidence, which highlighted the need for more rigor in standardizing methods and analytic decisions across studies. All studies included in this review (as well as the parent study [ADNI] used for the empirical follow-up) were cross-sectional at the point of cut-off determination, and longitudinal within-subject change will be an important direction for future studies to further improve rigor in the derivation of cut-points. Furthermore, several studies used overlapping samples which can lead to bias in our results. Finally, the use of MMSE and memory recall as outcome variables may have resulted in circularity with cut-points that were derived based on cognitive diagnosis. However, the specific neuropsychological measures used to determine diagnosis were independent from the cognitive outcomes used, and it is noteworthy that effect sizes for cognitive outcomes were commensurate with CSF outcomes. Additionally, the inclusion of cognitive outcomes to assess concurrent validity is critical given this is what we ultimately want to use biomarkers to predict and treat.

Importantly, the studies reviewed are further limited by the significant lack of diversity in race/ethnicity in tau PET research specifically and AD research in general. This is a significant limitation of our empirical follow-up as well, which was conducted in a predominately White, highly educated ADNI sample. The presence and degree of biomarkers such as tau may vary across racial/ethnic identities [52] as a result of numerous sociocultural factors including experience of racism and access to resources [53]. Exploring these and other moderating variables is essential to gain a holistic understanding of tau PET and the derivation of cut-points. Comparison of cut-point methods based on sample characteristics (e.g., community-based vs. cohort study) and demographics should be investigated in future studies. Indeed, deriving sex-specific and/or race-specific cut-points may be warranted given differences in biomarker levels across different groups.

## Conclusions

This review and empirical examination of tau PET cut-point methodologies demonstrates the significant heterogeneity in methods used to derive tau PET cut-points with its most widely studied tracer to date, Flortaucipir, resulting in a large range of suggested cut-points across studies. Factors influencing this variability may include differences in sample characteristics, reference region, use of PVC, region(s) of interest, or analytic procedures. This variability in tau PET cut-points has significant implications for increasingly used biomarker classifications that rely on these cut-points for determination of biomarker positivity and for potential selection for clinical treatment trials, as well as increasing estimation uncertainty which hinders reproducibility in AD biomarker research. Our empirical follow-up systematically addressed how these decisional and analytic choices in the methods used influenced cut-points and resultant positivity rates in a single sample of participants. Taken together, this paper highlights the importance of careful selection of thresholding methods based on the specific research goal to create and apply reliable and optimal cut-points that improve our characterization of AD biomarker risk.

## Data Availability

Data used in this study are available through the Alzheimer’s Disease Neuroimaging Initiative and can be found at http://adni.loni.usc.edu/.
